# Electroplating Cobalt Films on Silicon Nanostructures for Sensing Molecules

**DOI:** 10.3390/molecules27238440

**Published:** 2022-12-02

**Authors:** Chihyang Chen, Zhe Kan, Zibo Wang, Haibin Huo, Mengyan Shen

**Affiliations:** 1 Department of Physics and Applied Physics, University of Massachusetts Lowell, Lowell, MA 01854, USA; 2 International Institute for Urban Systems Engineering, Southeast University, Nanjing 210096, China

**Keywords:** nanostructure, electroplating, CoO, gas sensor, CO, and CO_2_

## Abstract

In this study, we electroplated Co and Cu on nano-spiked silicon substrates that were treated with femtosecond laser irradiations. With energy-dispersive X-ray (EDX) analysis by a scanning electron microscope (SEM), it was found that both Co and Cu are primarily coated on the spike surfaces without changing the morphology of the nanospikes. We also found that nanoscale bridges were formed, connecting the Co-coated silicon spikes. The formation of these bridges was studied and optimized through a series of time-controlled electroplating and oxidizing processes. The bridges are related to the oxidation of Co in the air. When it is irradiated with visible light, this special structure has shown a capability of interactions with carbon monoxide and carbon dioxide molecules. The electroplated cobalt may be used for gas sensors.

## 1. Introduction

Nanofabrication has been a leading study area in the development of material engineering. Regular nanostructures have been fabricated and studied on substrates of various materials by using femtosecond laser irradiation methods [1,2,3,4,5]. Previous studies of the formation of the nanostructures have revealed the mechanisms of refraction of laser light in highly excited silicon, interference between scattered light and refracted light, rapid cooling in liquid, roughness-enhanced optical absorption, and capillary instabilities of molten thin layers [4,5]. Some of these femtosecond-laser-nanofabricated materials have been utilized for surface-enhanced Raman scattering (SERS), field emission, and catalytic chemical reaction, as well as gas sensing [2,6,7,8,9]. Femtosecond laser irradiation was used to make composites with a layer of cobalt monoxide (CoO) nano-flakes on which some gold (Au) nanoparticles were deposited. The composite worked as a photo-catalyst to convert the carbon dioxide (CO_2_) and water (H_2_O) to methanol (CH_3_OH) [9]. Experiments using ^13^C-labeled CO_2_ and theoretical calculations have revealed strong CO_2_ adsorption onto the nanostructured Co/CoO surface of the catalyst [8]. The photon energy of visible light is large enough to excite the adsorbed CO_2_ molecules into an unstable state that reacts with dissociated H_2_O to form long-chain hydrocarbons because of the CO_2_ adsorption from air directly onto the CoO. The catalyst does not require purified CO_2_, and thus enables the low-cost production of diesel-range hydrocarbons with solar energy [8]. These nanostructures are also found to be applicable in gas sensing. Studies have shown that the high area/volume ratio and sharp structures of the nanospikes significantly enhance the sensitivity of SnO_2_ at room temperature [7,10,11,12,13,14,15]. In this work, we use silicon substrates with nanostructures generated by femtosecond laser irradiation to coat other materials for applications, for example in gas sensing.

Electroplating can fabricate large areas of cost-effective metal layers [16,17]. A previous study successfully electroplated cobalt on n-type silicon with special advantages or functions on application [18]. Co thin films deposited on n-type Si (111) wafers from Watts bath at pH 4.2 were characterized by cyclic voltammetry, chronoamperometry, atomic force microscopy, X-ray diffraction, and alternating gradient field magnetometer measurements, showing potential applications [18].

We implement femtosecond laser-induced silicon nanospikes as the substrate for electroplating metals. Cu and Co are selected as coating candidates as Cu is generally used for demonstrating electroplating and Co may have more applications. Current density and coating duration are used as parameters to actively control the process of electroplating. The samples are then further characterized with energy-dispersive X-ray (EDX) analysis using a scanning electron microscope (SEM), and with focused ion beam (FIB) milling. The SEM shows that interesting nanostructures grow under ambient air after electroplating. The EDX analysis shows that those new nanostructures primarily consist of coated metal and oxygen. FIB-SEM is conducted to confirm the tightness between the silicon substrate and coated metal.

The metal oxide nanostructures generated by this method may be used for sensing gases [7,10,11,12,13,14,15]. We studied the electrical response of the electroplated Co on nanostructured silicon substrates to gas molecules. The sample is placed in a chamber with different gases to test the relation between its electrical current and time. Nitrogen (N_2_), carbon monoxide (CO), and CO_2_ gases were tested. The Co/CoO on the silicon nanostructures demonstrated a capability of sensing gases for carbon monoxide as well as carbon dioxide. The sensing ability is obviously enhanced by white light illumination on the nanostructures. The enhancement is associated with the interaction between gas molecules on the surface and the photocarriers in the nanostructures. The enhancement will play an important role in collecting very weak signals of low concentration gases with a lock-in technique in the future [19]. The electroplated cobalt can be developed as a gas sensor, especially for sensing CO_2_.

## 2. Methodology

**Figure 1:**
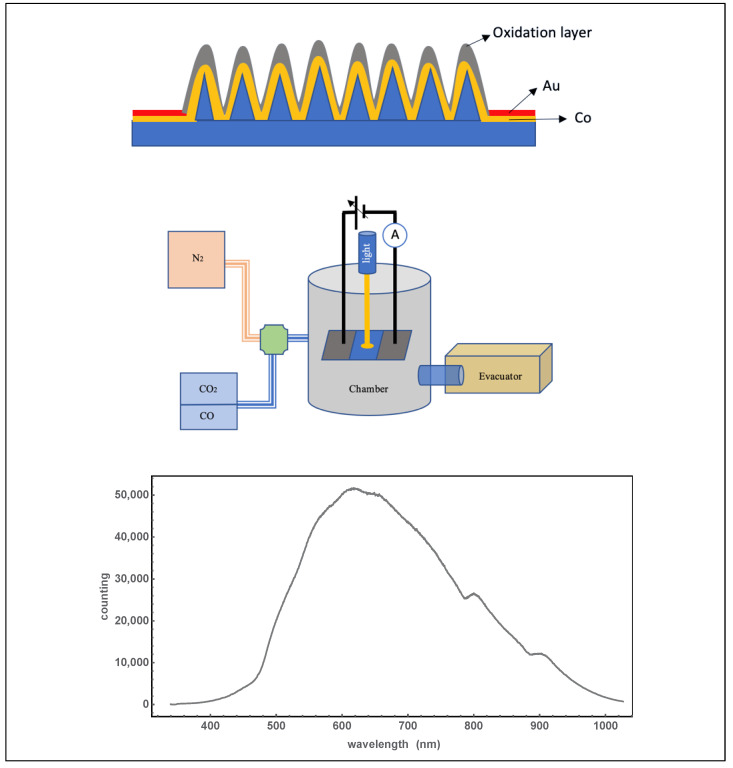
Sensing gases with CoO on the nanostructured Si surface. Upper: A sketch of the gas sensing sample by using the Si surface, electroplating of Co, sputtering gold for electrodes, and oxidation for 24 hours (the sample’s photo is given in Figure S9 in the Supplementary Materials). Middle: Gas sensing experiment settles with different gases (the setup’s photo is given in in the Supplementary Materials). Lower: Spectrum of the illumination light in the experiments.

Si nanospikes were obtained using the femtosecond laser irradiation method on a p-type silicon substrate [2,3,4,5], although the nanospike formation does not obviously depend on the type of silicon. The resistivity of a typical p-type substrate is low enough for electroplating metal onto the substrate. A chronoamperometry method from VersaSTAT 3 Potentiostat Galvanostat (Princeton Applied Research, Oak Ridge, TN, USA) was selected to control voltage and current during the electroplating. CuSO_4_ and CoCl_2_ were selected as electrolyte solutions for coating Cu and Co, respectively. The anode metals were 99.99% pure cobalt or copper foils. SEM and EDX were conducted through JEOL JSM 7401F. FIB was conducted through Zeiss Auriga for further analysis of the nanostructures. SEM showed that bridge-like nanostructures formed, and EDX confirmed that the bridges are of Co and O. Large numbers of Co coated on p-type silicon wafers with an effective region of 1 cm × 1 cm were prepared for samples of detecting gas molecules. The area without nanostructures was used to make electrodes. Gold electrodes were made with a vacuum sputter coater (Denton Vacuum Desk IV, Moorestown, NJ, USA) by covering the area of none-structured region as shown in Figure 1. The thickness of the gold electrodes was estimated to be about 200 nm. When all the steps were completed, the edge and the bottom of the wafer were grinded, and the sample was oxidized for 24 hours in the air and at room temperature.

**Figure 2:**
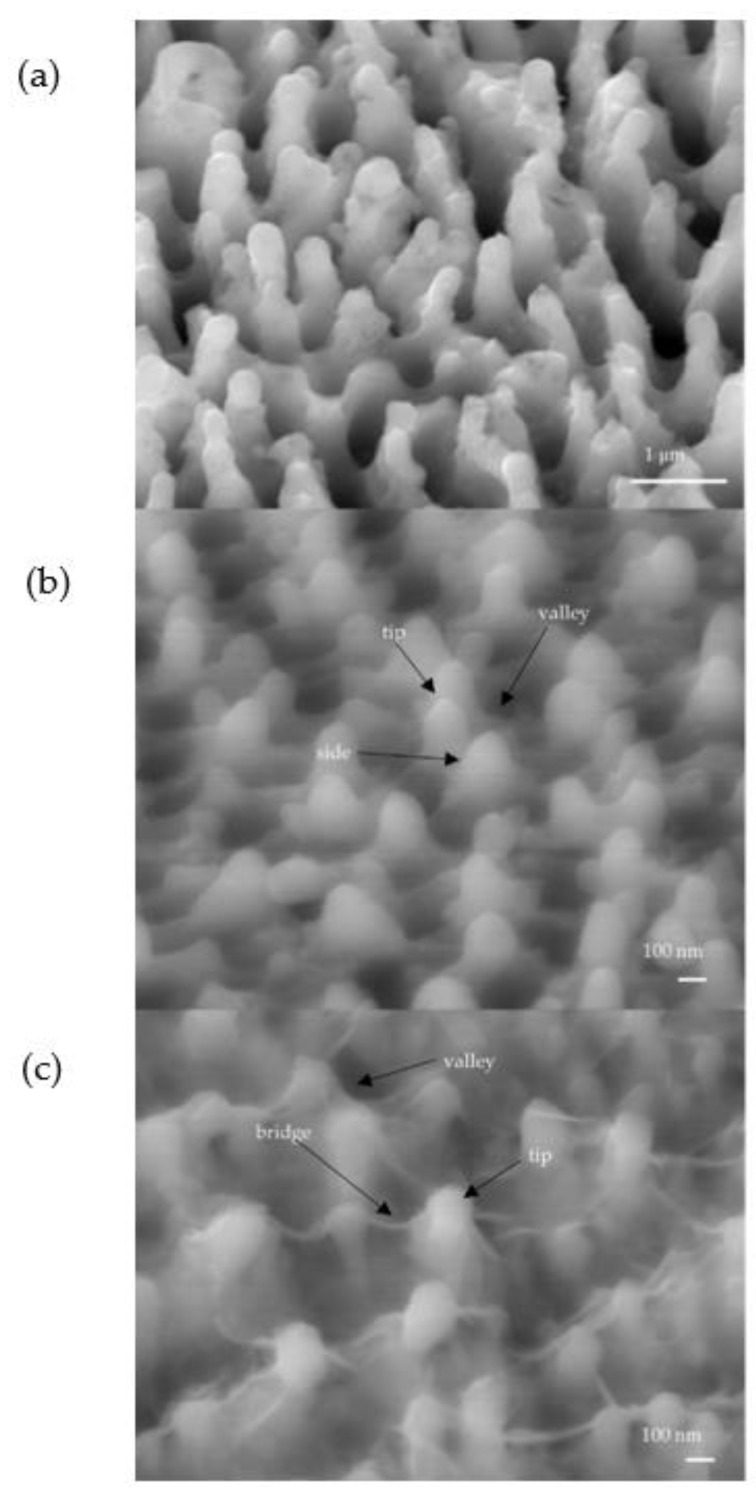
SEM images of silicon with nanospikes coated with different metals: (**a**) before electroplating; (**b**) after Cu electroplating; (**c**) after Co electroplating.

**Figure 3:**
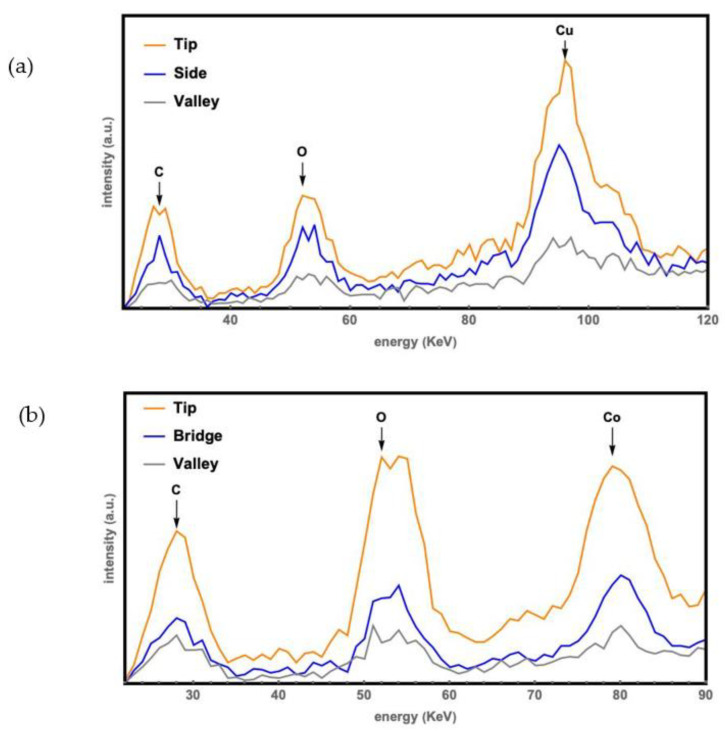
Energy-dispersive X-ray analysis. Upper (**a**): of silicon wafer with nanospikes coated by Cu; lower (**b**): of silicon wafer with nanospikes coated by Co.

After the sample was prepared as shown in Figure 1, the gold electrodes were welded with wires and put into a chamber. [An asymmetrical electric circuit (Supplementary Materials Section S1) showed that the main electric current flows through the CoO/Co layer. The gas response curves of the samples were obtained through electrical measurement with an applied voltage of 2.5 V at room temperature, as shown in Figure 2. After the sample was placed in the chamber, it was evacuated by a mechanical pump under 1.5 pascal, which can pump in N_2_ to an atmospheric pressure. Then, the chamber was filled with N_2_ gas to an atmospheric pressure and stabilized for more than 15 minutes. This purification was performed three times to eliminate any contamination. A CO_2_ gas tank with 99.999% purity was connected to the chamber with a regulated flow valve. A tank of (CO + N_2_) gas with a CO concentration of 92.04 PPM (Mole %) was also connected to the chamber with a regulated flow valve. Light from a lamp was waveguided to the spiked area of the sample surface. The spectrum of the light is shown in Figure 3. and the intensity of light was about 100 μW/mm^2^.

## 3. Results and Discussion

### 3.1. Sample Preparation

SEM images of silicon spikes before and after electroplating are shown in Figure 2. The SEM image in Figure 2b showed that Cu could be uniformly coated on Si spikes; however, there is a tendency to form bridge structures among Co-coated Si spikes as shown in Figure 2c. From these SEM images, it was found that the metal also covers the valleys between the spikes after electroplating. The size of silicon nanospikes was measured and analyzed over these three samples: for the wafer without coating, the average spike height is 942 nm; for the wafer coated by Cu, the average spike height is 318 nm; and for the coated Co sample, the average spike height is 424 nm. We observed that the Co coating forms a spider-web structure between spikes and covers the whole wafer. This is very different from that of Cu where no unusual structure is found. Hence, only the Co coating is studied in this work. In the EDX analysis data, there was carbon (C), cobalt (Co), and oxygen (O) for the bridge structure in the electroplated Co sample.

A series of time-controlled oxidation was conducted and summarized in 

Table 1; 20 hours was found to be sufficient for bridges that connect each spike to form. The EDX analysis showed that the bridge structures consist of Co and O, which supports the assumption that the bridge results from the oxidation of Co. Because the oxidation of Co in the air at room temperature synthesizes CoO, the bridge structures consist of CoO [8].

The EDX data in Figure 3 are normalized by the original silicon wafer with nanospikes without further coating. The oxidation level was found to be positively related to the thickness of coated metal. Furthermore, the amount of coated metal on the tips was found to be thicker than that at its surroundings. The oxidation level was found to be the highest on the tip.

Moreover, we found that the Si spikes enhance the amount of electroplating metal in comparison to the amount of electroplating metal on a smooth Si surface. It is much more difficult to electroplate metal uniformly on a smooth substrate than on a nanospiked substrate. The SEM with EDX analysis also shows that cobalt was hardly coated on a perfectly smooth region; only large cobalt dust accumulated on the smooth substrate. However, on nanospiked substrates, the cobalt could be coated more evenly on the silicon wafer by electroplating. Therefore, the nanostructure enhances the electroplating efficiency due to possible electric field enhancement on the tip of silicon. 

**Figure 4:**
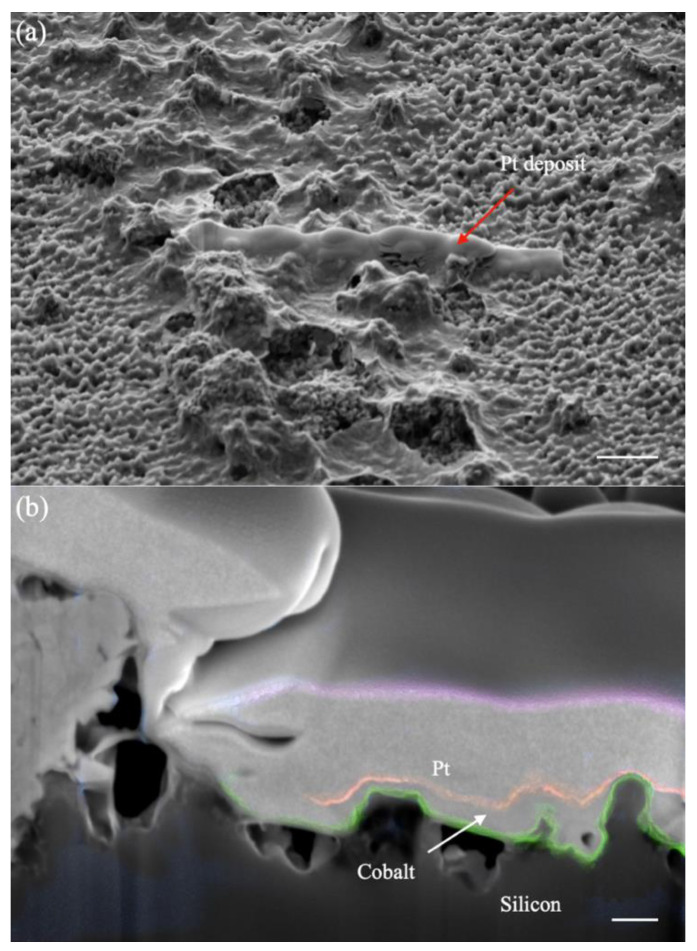
SEM images of the sample. (**a**) The Pt deposited part was selected for FIB milling (scale bar 3 μm). (**b**) SEM image at a 52° angle view after the FIB milling (scale bar 200 nm). The area with the nanospikes is identified as Si, and its boundary between Co (shown in green color) is recognized with silicon’s dark and cobalt’s gray colors; the subsequent layers were then identified as the coated Co and the coated Pt for FIB milling. The boundary between Co and Pt (shown in orange color) is clearly shown with their uniformity. Co coating is uniform while the Pt is not uniform.

FIB was used to cut the spike and study the thickness of coated metal as shown in Figure 4. In Figure 4a, the Pt deposit was the target place cut by FIB and that area was filled with a lot of dust. Figure 4b represents the side view of the cut area, and every boundary was marked by a different color to separate it. The boundary of the silicon and cobalt layer was labeled with a green color. The boundary of deposited Pt and cobalt was labeled with an orange color. Figure 4b shows that the cobalt layer of the tips was not the thickest part on the side view as concluded with the EDX analysis. The difference may originate from the mechanism of the oxidation of Co on the surface, but this needs further study. Figure 4b also reveals that the silicon spikes or pillars are in the same body with the silicon substrate. This property is very important to develop the nanostructures for potential applications. 

**Figure 5:**
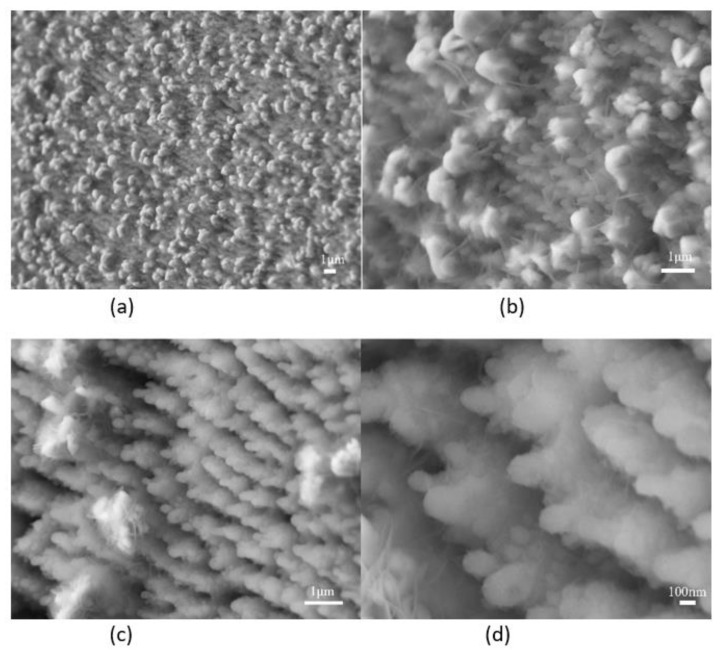
Coated large Co on the tip to cover the nanospike structures; upper: at high current density with different SEM resolutions (with low (**a**) and high (**b**)); lower: at low current density with different SEM resolutions (with low (**c**) and high (**d**)).

According to previous studies, the nanostructured Si-Co substrate could be further modified as a gas sensing device because gases, such as CO_2_, are easily adsorbed on the CoO surfaces [7,8,9]. Under different voltages, the current density changed by electroplating different thicknesses of metal. Under the high-energy situation, SEM showed that the metal crystal would grow up like the dust on the spike tip and cover the surrounding area of nanospikes. In Figure 5, we can observe a lot of huge metal dust built on the tip of the nanospikes. At the current density 3 times less than the high-energy situation which was shown in Table 2, SEM images showed the electroplating metal Co grew on the tip but not overcoated on the surface as shown in Figure 5. Additionally, EDX showed that the value of oxidation is related to the thickness of coated metal Co.

**Figure 6:**
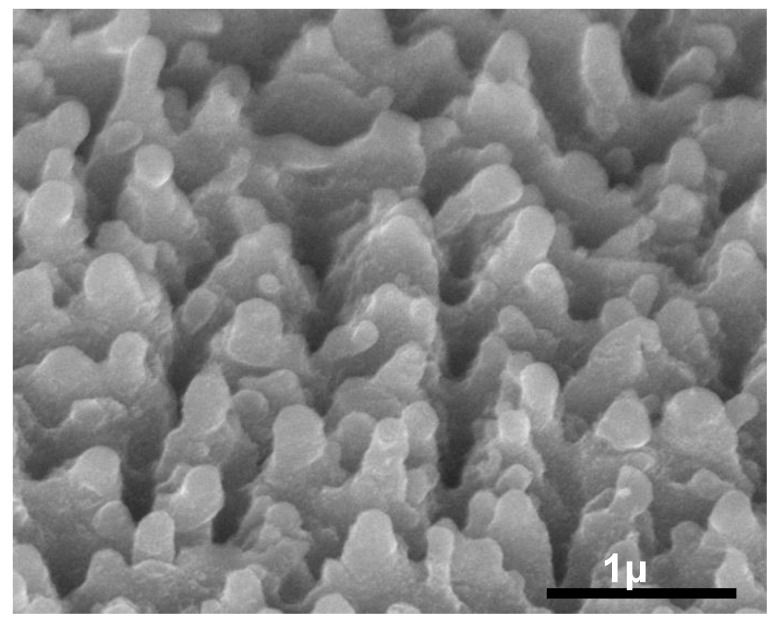
Intrinsic silicon wafer after electroplating cobalt.

Intrinsic silicon wafers have been attempted for electroplating cobalt; however, the SEM images show that the shape of the structures remains unchanged as shown in Figure 6, and EDX analysis showed that the cobalt was hardly coated. Low resistivity of p-type or n-type silicon is necessary for the electroplating. 

### 3.2. Interactions between Molecules and the Electroplated Cobalt under Illumination

**Figure 7:**
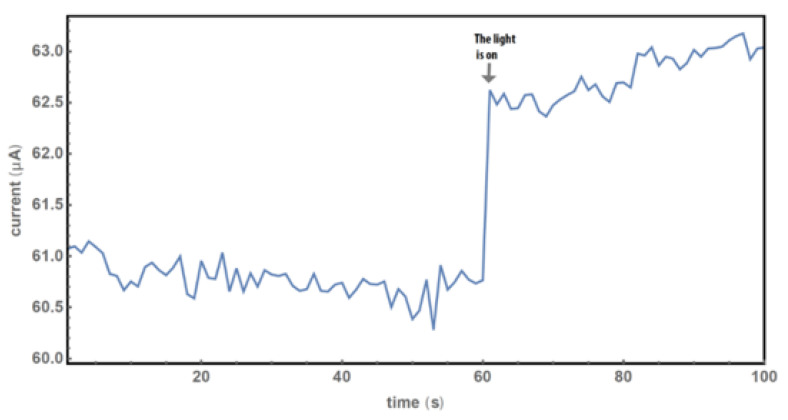
Photocurrent of the sample. The intensity of light was about 100 μW/mm^2^ and the area was 1 cm^2^ on the sensor.

With the setup in Figure 1, we found that the electric current of the sample responds to the injection of CO_2_ or (CO + N_2_) gases when the silicon wafer with Co-coated nanospikes interacts with light. When light was illuminated on the sample, the current increase was observed (Figure 7), and the response time was less than 0.001 seconds, as shown in Figure S7. It was shown that the photocurrent greatly enhances the sample’s sensitivity (comparing the results in the Supplementary Materials Section S2 with those in the following section). We will concentrate on the study of the gas sensing under visible light illumination because the enhancement plays an important role in collecting very weak signals of low concentration gases with a lock-in technique in the future [19]. 

### 3.3. Test for Intereactions between Molecults and the CoO Surface 

**Figure 8:**
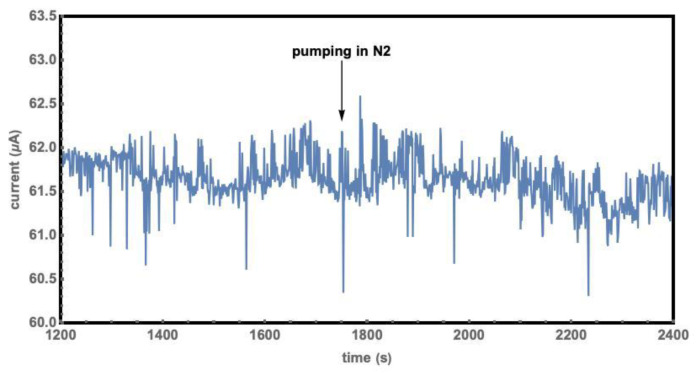
I-t curve of filling N_2_ into a chamber which was pre- evacuated to 1.5 Pa.

The chamber was evacuated to 1.5 Pa before being filled in with gas, namely N_2_, (CO + N_2_), or CO_2_. The voltage applied to the sample was actively held at 2.5 V by the VERSA station. Nitrogen gas was released into the chamber 20 minutes after evacuation, to a pressure of one atmosphere in a few seconds, and no obvious current change had been found after another 10 minutes, as the results show in Figure 8. Therefore, nitrogen was selected as a background and was used as a purge gas since the sample did not respond to nitrogen. 

**Figure 9:**
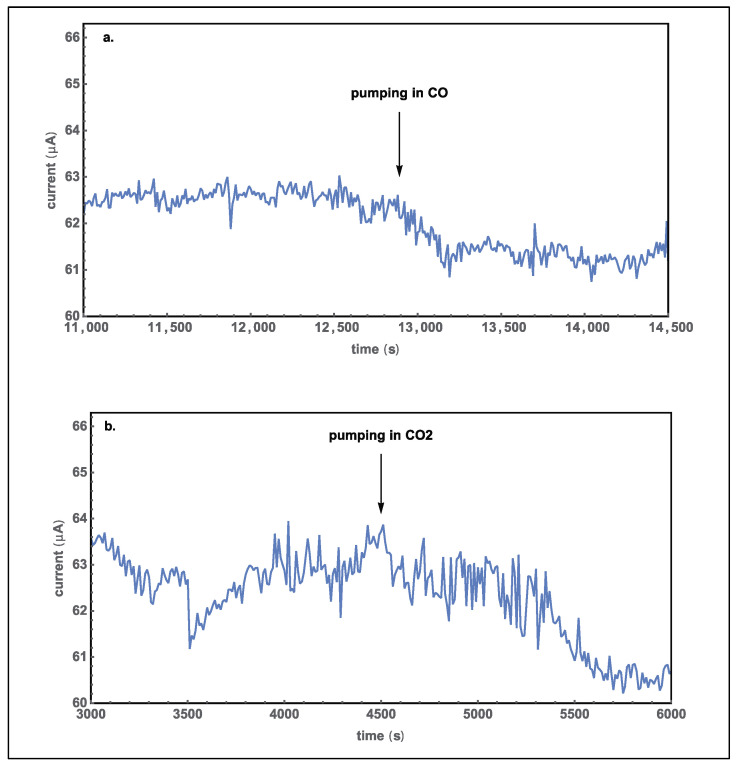
The current versus time graphs when the chamber was pre-evacuated to 1.5 Pa. (**a**) For filling in (CO + N_2_) and (**b**) for filling in CO_2_.

Similar experiments were also done for (CO + N_2_) and CO_2_ for measuring the electric currents through the sample when the respective gas was released into the chamber from vacuum to 1 atm in a few seconds. The currents of (CO + N_2_) and CO_2_ change more obviously than that of nitrogen. The current decreased when (CO + N_2_) or CO_2_ was pumped into the chamber, as indicated by the arrow, as shown in Figure 9. There is a 3 μA decrease after filling in CO_2_ for 1500 seconds. There is a 1.5 μA decrease after the chamber was filled with (CO + N_2_); the small change results from the low concentration of CO in N_2_, at 92.04 PPM (Mole %).

**Figure 10:**
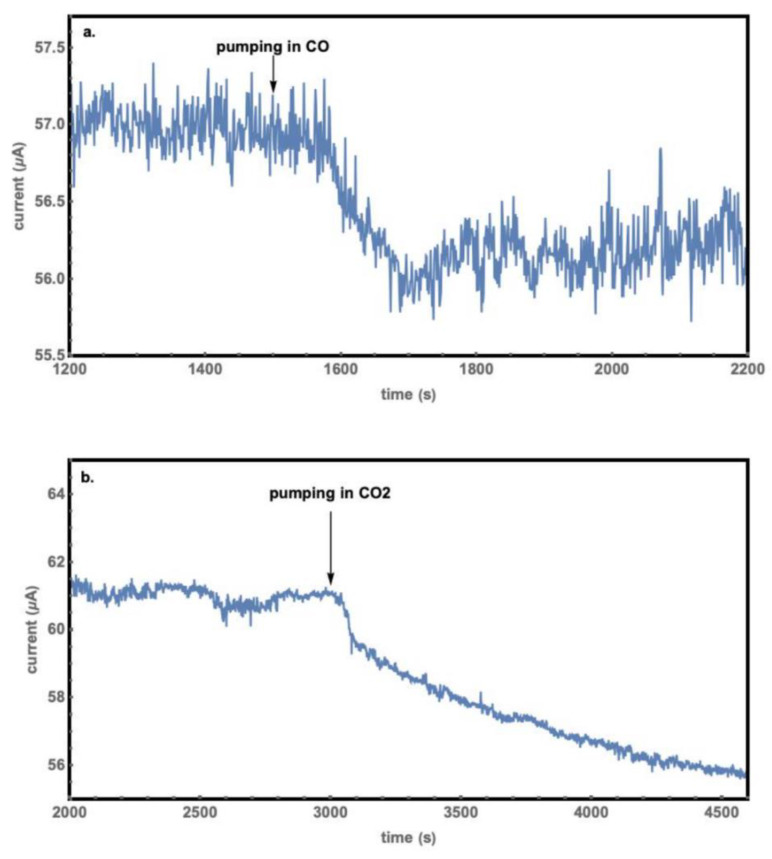
I-t curve of the sample in the nitrogen pre-filled chamber. (**a**) For further filling in of (CO + N_2_) and (**b**) of CO_2_.

To further study the response of the sample to CO and CO_2,_ we first set the pressure of three gas tanks (pure N_2_, pure CO_2_, and (CO + N_2_)) to 1 atm; second, we purged the sample in the chamber with nitrogen three times and closed the pumping system; third, we filled the chamber with pure nitrogen gas to about 0.9 atm; and fourth, we opened the valve between the chamber and special gas so that a small amount of the special gas filled into the chamber. During these steps, the electric current was recorded, and we observed the change after releasing the special gas into the chamber. The results are shown in Figure 10. The electric currents of (CO + N_2_) and CO_2_ still had an obvious decrease when nitrogen had been in the chamber. Figure 10a shows that the current decreased by 1 μA after CO was released into the chamber. The current for CO_2_ also decreased, as shown in Figure 10b. The decrease of the current was 6 μA after CO_2_ gas was filled in for 15 minutes. These results show that both CO_2_ and CO interact with the sample at lower concentrations. The decrease of current led to the conclusion that the electrical conductivity of the CoO nanostructures decreased upon injection of CO_2_ or CO gas, which related to their adsorption onto the CoO surface [8,20].

### 3.4. The Mechanism of the Interaction between Molecules with the Electroplated Cobalt Surface

Metal oxide gas sensors usually work at high temperatures (>150 °C). Adsorbed oxygen molecules play the main role in the sensing mechanism [7]. Electrons may transfer from the material to the oxygen, and the oxygen molecules will be in the atomic (O^2−^) or molecular ($O_{2}^{-}$) forms; then, analyte molecules will be adsorbed and react with the oxygen molecules at very high temperatures, and electrons will transfer back to the semiconductor and the reaction product may leave from the sensor surfaces. The mechanism is not suitable for the sensing signal in this work because the working temperature is at room temperature. The surface enhancement and strong local electric fields of nanostructures are associated with the sensing signal in this work [7]. 

**Figure 11:**
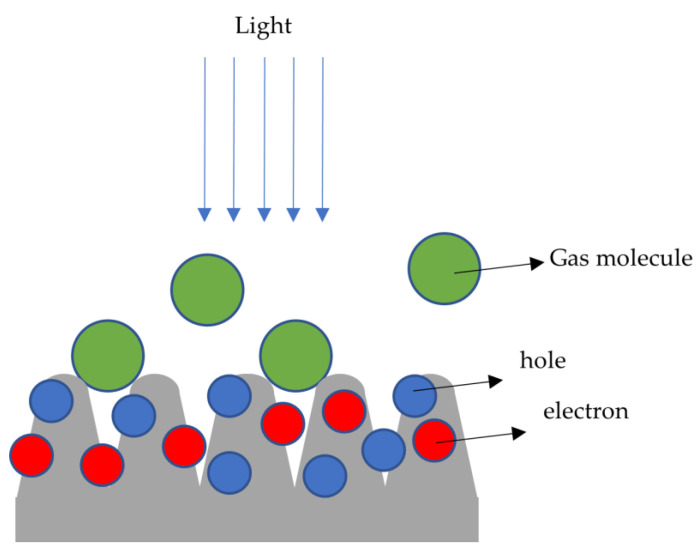
After light illumination, the gas molecules would have more chance to interact with the electrons and holes.

The experimental results have shown that when CO and CO_2_ molecules interact with the CoO surfaces, the current decreases. The light illumination on the CoO surfaces is necessary to have the obvious current decrease. The total electric current is proportional to the electric conductivity σ of the sample. If the CoO semiconductor has both electrons and holes, the total conductivity is:

(1) $$\sigma =e\left({n\mu }_{e}+{p\mu }_{h}\right)$$

where n and μ_e_, and p and μ_h_ are the concentrations and mobilities of electrons and holes, respectively [21]. When there is no light illumination on the CoO surface, the CO and CO_2_ molecules cannot obviously change the current or the conductivity. Almost all the electrons and holes are in the body of the conductors when there is no light illumination, which results in a weak interaction between the body carriers and the gas molecules on the surfaces. While light is illuminated on the CoO surfaces, after a photon is absorbed when the photon energy is larger than the band gap energy of CoO or equal to the binding energy of a donor or an acceptor, free carriers are generated at the surface of CoO (n and p increase) as shown in Figure 11, thus the current increases. Further experimental data of the wavelength dependence of the photocurrent are needed to test this model. The photocarriers on the CoO surface have more chances to interact with the CO and CO_2_ molecules [22,23]. Because of the following:

(2) $$\mu =\frac{e}{m}\tau$$

where *m* is the carrier effective mass and *τ* is the average scattering time, the interaction leads to a shorter average scattering time. The interaction results in the decrease of the mobilities on the CoO surfaces, thus the photocurrent decreases as shown in Figure 9 and Figure 10 when CO or CO_2_ molecules are filled into the chamber. Sensing CO molecules have been intensively studied [7,10,11,12,13,14,15], while sensing CO_2_ molecules with semiconductors has seldom been investigated because of their weak interactions with semiconductor surfaces. The observation of light-enhanced CoO’s response to CO_2_ molecules is in agreement with the strong adsorption of CO_2_ onto the CoO surface, and the adsorption makes an artificial photosynthesis possible [8]. Further detailed studies are necessary to know the sensitivity, selectivity, response and recovery time, detection limit, stability, and light irradiation conditions for making a CoO gas sensor.

## 4. Conclusions

Electroplated Co on a silicon wafer with silicon nanospikes forms bridge nanostructures among the nanospikes. The bridge structure formation is related to the oxidation of Co in the air at room temperature. It was found that after about 24 hours of oxidation, uniform numbers of bridge structures form among the nanospikes. With the new CoO bridge structures among the nanospikes, we produced a sample to detect different gases. The sample responds well to CO as well as CO_2_ molecules. It was found that when a white light illuminates on the sample, the electrical current increases and responds to light in a time less than one second. The photocurrent greatly enhances the sample’s sensitivity. As far as we know, in previous publications, there are no such results where light illumination greatly increase the CO_2_ sensing signals with semiconductors, although there are similar results for CO in previous publications [22,24]. The enhancement plays an important role in collecting very weak signals of low-concentration gases with a lock-in technique in the future [19], suggesting that it is important to further study the properties for future use of the CoO structure as a gas sensor.

## Figures and Tables

**Table 1:** The number of Co bridges between Si spikes in an area of 3.5 × 10^6^ nm^2^ with different oxidation times.

	Current Density	Oxidation Time	Bridge Number
1st	0.5μA/cm2	24 Hours	26
2nd	0.5μA/cm2	3 Hours	6
3rd	0.5μA/cm2	0.5 Hour	7

**Table 2:** The current density of electroplated Co on the nanospikes Si wafer in different situations.

	Figure 4	Figure 5
Current Density	4.55 μA/cm^2^	1.48 μA/cm^2^
Numbers of Dust per Area (150 um^2^)	27	5

## Data Availability

Not applicable.

## Supplementary Materials

The following supporting information can be downloaded at: https://www.mdpi.com/article/10.3390/molecules27238440/s1, Figure S1. I-V curve of sensor sample just after electroplating; Figure S2. The electroplating experiment settles with the electrolyte; Figure S3. The asymmetry structure of the sample. The dotted lines represent the paths of current; Figure S4. The previous sample and new sample by different positions of electrodes; Figure S5. I-V curve of the sample in the chamber with CO_2_ in this work; Figure S6. I-t curve by pumping in special gases without light illumination; Figure S7. The responses of photocurrent when the light is on/off; Figure S8. Photos of gas sensing set up (see Figure 1). The Versastat station provides a stable voltage at 2.5 V, and the current flowing in the circuit is continuously recorded in a computer with a Versastat software. Light from a regular illumination bulb (whose spectrum is shown in Figure 1) through the window will be used for gas sensing signal enhancement; Figure S9. The fabricated gas sensing sample. Upper: the sample testing in air. Lower: the sample installed in a vacuum chamber sensing measurement.

## Sample Availability

Samples of the compounds are available from the authors.
