# Supplementary material for: Electroplating Cobalt Films on Silicon Nanostructures for Sensing Molecules
Source: Molecules. 2022 Dec 2;27(23):8440. doi: 10.3390/molecules27238440 (PMC9738422; doi:10.3390/molecules27238440)
Supplement: Supplementary file 1 [file molecules-27-08440-s001.zip › molecules-2041387-supplementary.pdf]

molecules

MDPI

Supplementary Materials

Electroplating Cobalt Films on Silicon Nanostructures for Sensing Molecules

Chihyang Chen[1], Zhe Kan[1], Zibo Wang[1], Haibin Huo[2] and Mengyan Shen [1,*]

1 Department of Physics and Applied Physics, University of Massachusetts Lowell, Lowell 01854, MA, USA
2 International Institute for Urban Systems Engineering, Southeast University, Nanjing 210096, China
* Correspondance : mengyan_shen@uml.edu (M.S.)

S1. The defects of sample

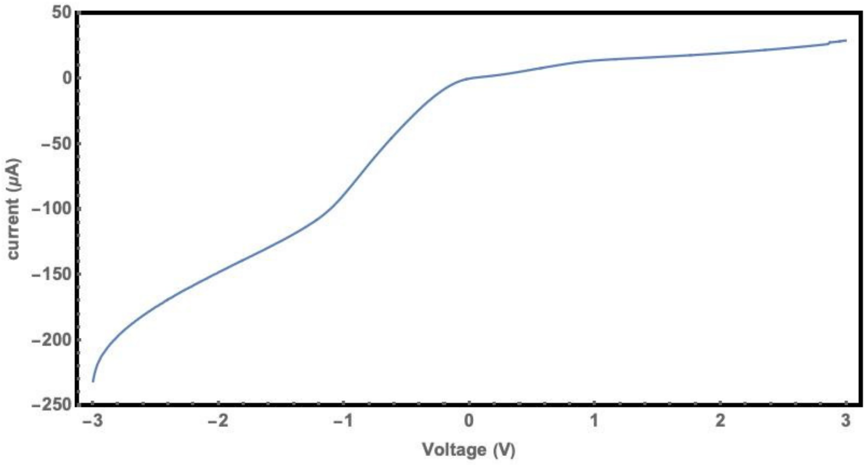

Figure S1. I-V curve of sensor sample just after electroplating.

After we finished the sample wafer first, we found that the I-V curve was not the symmetric curve from negative to a positive voltage, as shown in Figure S1. However, when we prepared to coat cobalt on the silicon, there must be one side to clip the electrode for electroplating as shown in **Figure 1**. The clipped side must be one small area in the air, and we know the sample must be under solution if we want to coat metal. Therefore, it would make a small area not be coated with cobalt on silicon. So, after electroplating cobalt and coated the gold for electrodes, there must be an unequal structure for two electrodes on the silicon wafer as shown in Figure S3. One side was the gold-cobalt-silicon structure. But there were two structures on the other side, the gold-silicon structure, and the gold-cobalt-silicon structure, and it could be the reason that causes the asymmetric structure.

The green line is the path of the current flowing through the Co layer. The red line is the path of the current flowing through p-type silicon. The green current path is symmetry, its I-V curve does not depend on the polarity of bias voltage. The red current path

is asymmetry. Because the Schottky barrier height of Co/p-silicon is about 0.4 eV [S1] and that of Co/p-silicon is about 0.3 eV, the resistance for the current flowing from the left to the right is smaller than that flowing from the right to the left. Therefore, its I-V curve depends on the polarity of bias voltage [S1, S2].

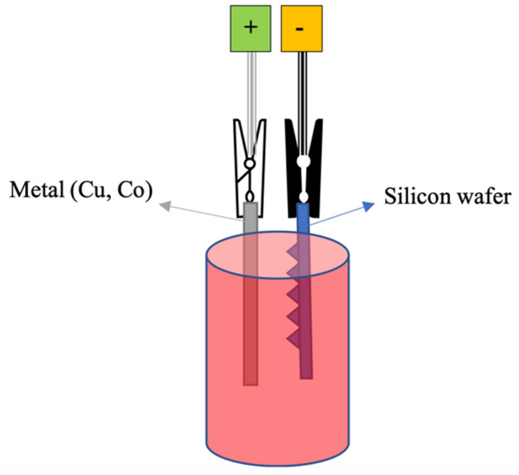

Figure 2. The electroplating experiment settles with the electrolyte.

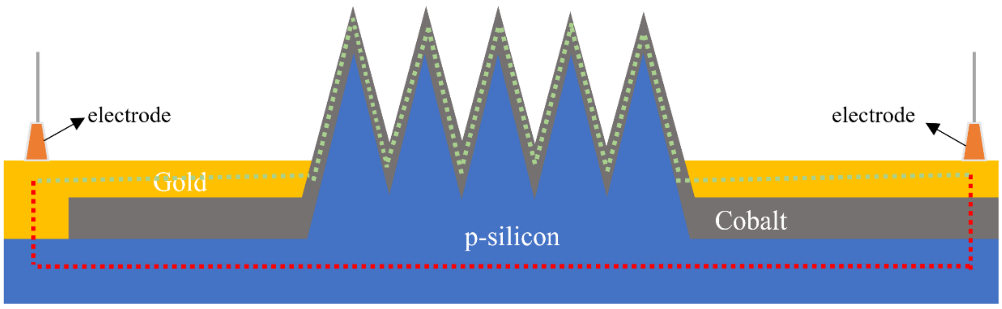

Figure 3. The asymmetry structure of the sample. The dotted lines represent the paths of current.

Therefore, we welded the electrode on the wafer at a different gold-cobalt-silicon surface. The new electrode is placed closer to the spiked area, as shown in **Figure S4**, and it could make sure that the current flows from gold to cobalt on the top surface. So, the current path of both sides would be the same and let the I-V curve become symmetry. We had the new symmetry I-V curve of a new sample when we tested the gas sensing part as shown in **Figure S5**. For our sample, we also wanted that the electron would pass through the top layer, which was the spike-structures coated with Co/CoO. Therefore, after replacing the electrode, the symmetric I-V curve could make sure for us that the current is mainly passing through CoO.

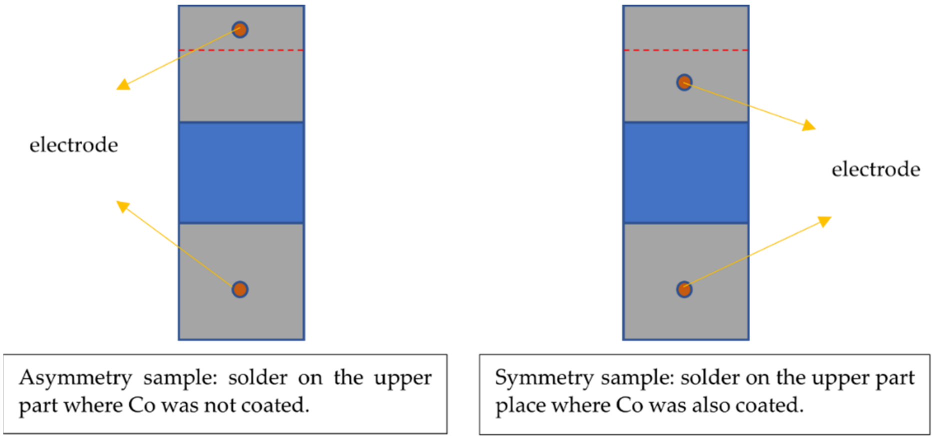

Figure S4. The previous sample and new sample by different positions of electrodes.

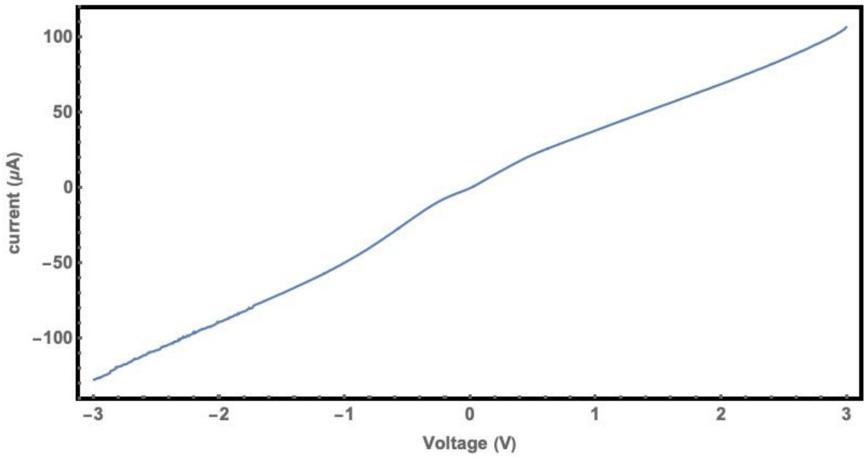

Figure S5. I-V curve of the sample in the chamber with CO_2 in this work.

S2. The signal without light illumination

The I-t curves after pumping in N_2, CO, and CO_2 without light illumination are shown in **Figure S6**. The currents for N_2 and CO don't show any obvious changes after pumping in the gases; and the current of CO_2 decreases only 0.5 μA after 10 minutes. The changes are much smaller than those in Figure 13. Light illumination is therefore enhancing the gas signal.

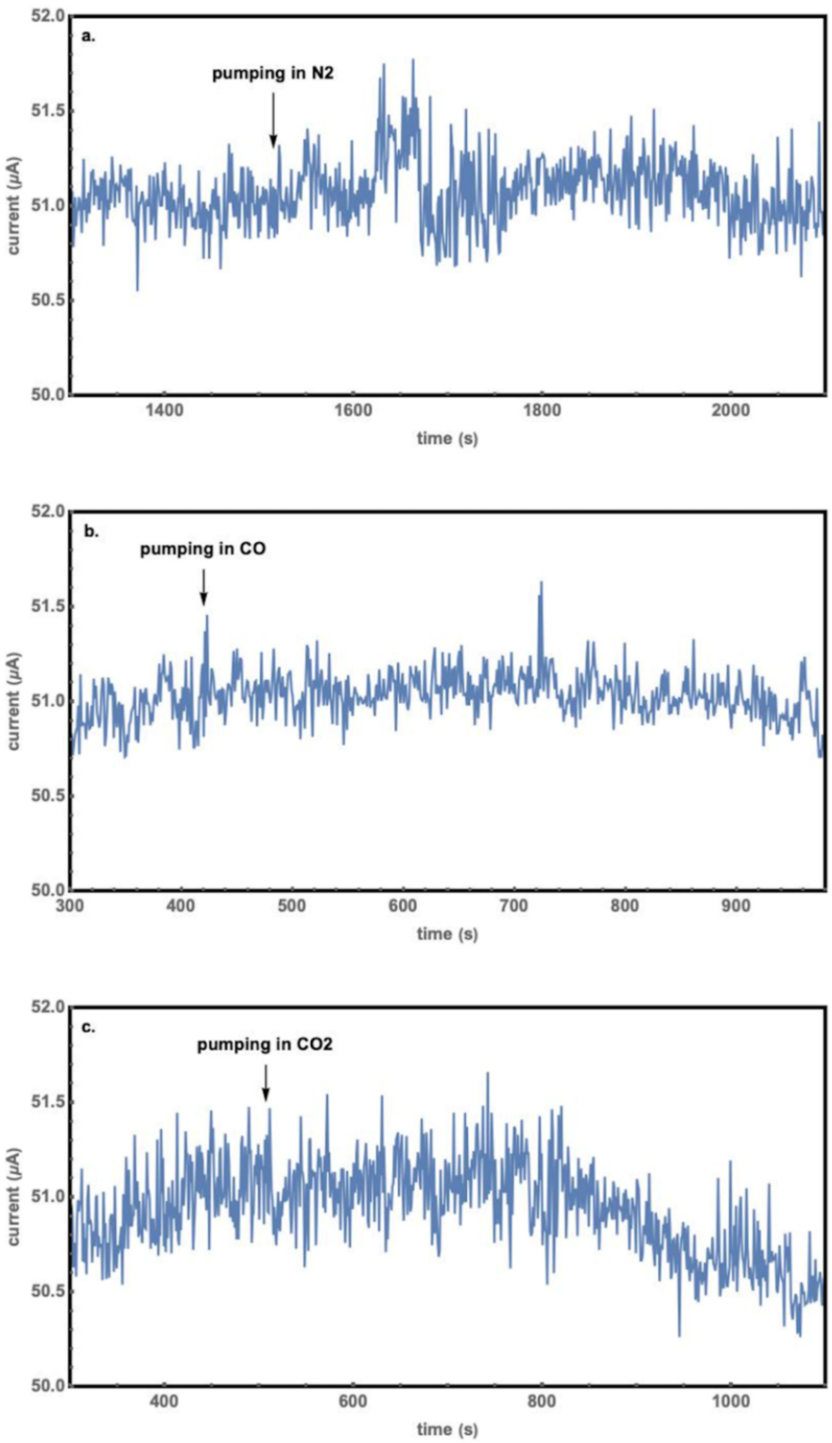

Figure S6. I-t curve by pumping in special gases without light illumination

S3. The responds time of light sensor

In the experiment, the response time of photocurrent observed to be less than 0.001 second as shown in **Figure S7**.

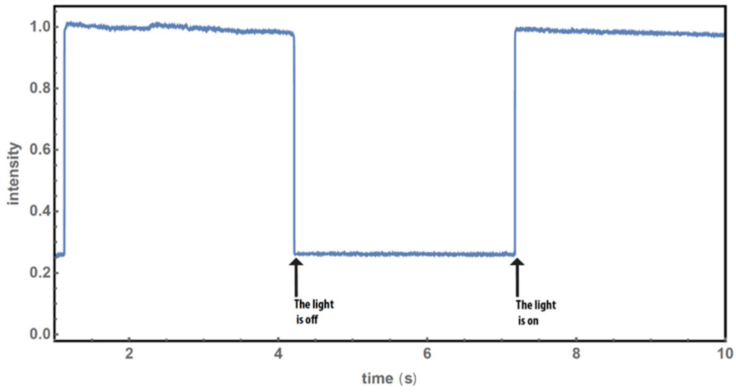

Figure S7. The responses of photocurrent when the light is on/off.

S4. Photos of sensing set up and the fabricated gas sensing sample

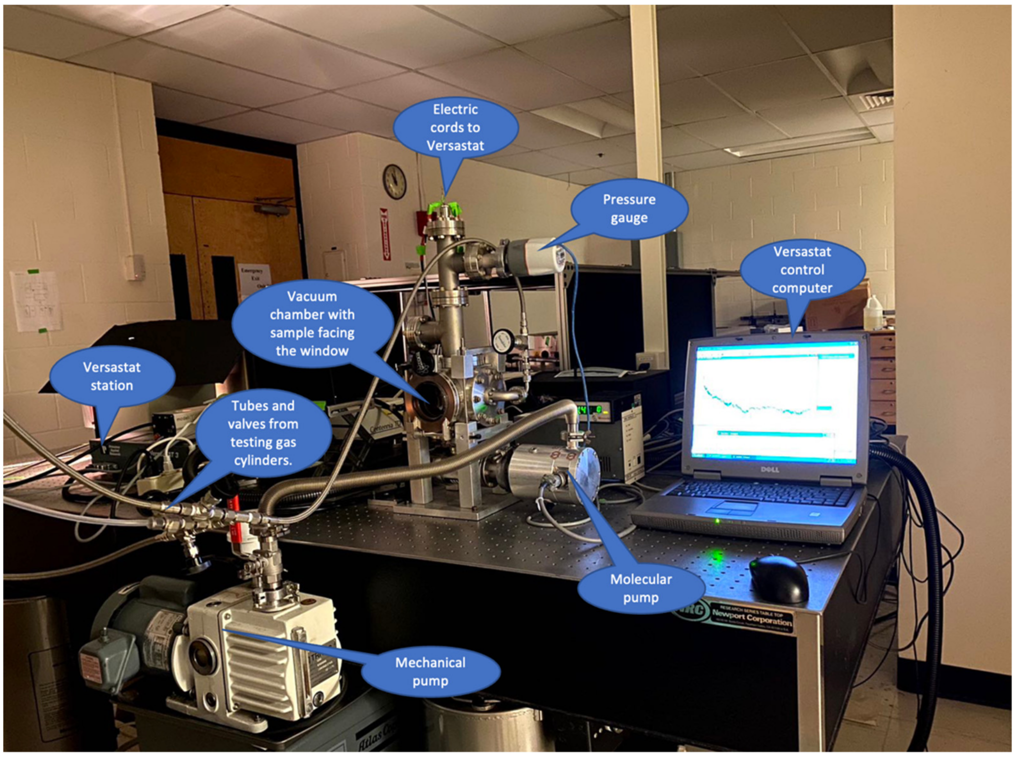

Figure S8. Photos of gas sensing set up (see Figure 1). The VersaStat station provides a stable voltage at 2.5 V, and the current flowing in the circuit is continuously recorded in a computer with a VersasSat software. Light from a regular illumination bulb (whose spectrum is shown in Figure 1) through the window will be used for gas sensing signal enhancement.

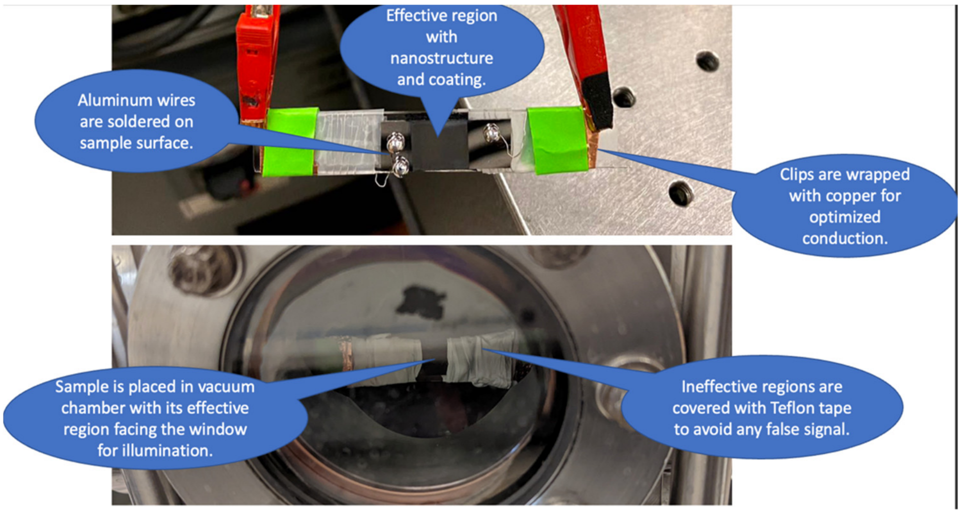

Figure S9. The fabricated gas sensing sample. Upper: the sample testing in air. Lower: the sample installed in a vacuum chamber sensing measurement.

References

S1. Van Gurp G. Cobalt silicide layers on Si. II. Schottky barrier height and contact resistivity. *Journal of Applied Physics* **1975**, *46*, 4308-4311.

S2. Balsano R, Matsubayashi A, LaBella VP. Schottky barrier height measurements of Cu/Si (001), Ag/Si (001), and Au/Si (001) interfaces utilizing ballistic electron emission microscopy and ballistic hole emission microscopy. *AIP Advances* **2013**, *3*, 112110.
